# Current Updates on Naturally Occurring Compounds Recognizing SARS-CoV-2 Druggable Targets

**DOI:** 10.3390/molecules26030632

**Published:** 2021-01-26

**Authors:** Isabella Romeo, Francesco Mesiti, Antonio Lupia, Stefano Alcaro

**Affiliations:** 1 Dipartimento di Scienze della Salute, Università “Magna Græcia” di Catanzaro, Campus “S. Venuta”, Viale Europa, 88100 Catanzaro, Italy; isabella.romeo@unicz.it; 2 Net4Science Academic Spin-Off, Università “Magna Græcia” di Catanzaro, Campus “S. Venuta”, Viale Europa, 88100 Catanzaro, Italy; francesco.mesiti@studenti.unicz.it (F.M.); antonio.lupia@net4science.com (A.L.)

**Keywords:** SARS-CoV-2, natural products, drug repurposing, multi-targeting, COVID-19, in silico, in vitro, in vivo, clinical trials

## Abstract

The severe acute respiratory syndrome coronavirus 2 (SARS-CoV-2) has been identified in China as the etiologic agent of the recent COVID-19 pandemic outbreak. Due to its high transmissibility, this virus quickly spread throughout the world, causing considerable health issues. The scientific community exerted noteworthy efforts to obtain therapeutic solutions for COVID-19, and new scientific networks were constituted. No certified drugs to efficiently inhibit the virus were identified, and the development of de-novo medicines requires approximately ten years of research. Therefore, the repurposing of natural products could be an effective strategy to handle SARS-CoV-2 infection. This review aims to update on current status of the natural occurring compounds recognizing SARS-CoV-2 druggable targets. Among the clinical trials actually recruited, some natural compounds are ongoing to examine their potential role to prevent and to treat the COVID-19 infection. Many natural scaffolds, including alkaloids, terpenes, flavonoids, and benzoquinones, were investigated by in-silico, in-vitro, and in-vivo approaches. Despite the large data set obtained by a computational approach, experimental evidences in most cases are not available. To fill this gap, further efforts to validate these results are required. We believe that an accurate investigation of naturally occurring compounds may provide insights for the potential treatment of COVID-19 patients.

## 1. Introduction

Coronaviruses are a diverse panel of viruses capable of producing infection in many animals and are responsible for respiratory infections in humans. After facing the fatal respiratory illness, caused by the Severe Acute Respiratory Syndrome Coronavirus (SARS-CoV) and the Middle East Respiratory Syndrome Coronavirus (MERS-CoV), the end of 2019 marked the start of one of the worse global pandemics [1]. More precisely, the first outbreak was beheld in Wuhan city, probably in the local seafood market, where there was witnessed a wave of noteworthy suspected pneumonia disease that began with progressive respiratory failure due to alveolar damage and even death. Successively, this “enemy” has been identified in SARS-CoV-2 as an etiologic agent of coronavirus disease 2019 [2]. SARS-CoV-2 is still a global health problem today. Due to its high transmissibility, the virus was able to spread quickly around the world [3].

On 11 March 2020, the World Health Organization (WHO) declared the coronavirus disease, namely COVID-19, a pandemic and the scientific community shifted its focus towards this fight. Today, there is no proven effective and specific treatment for COVID-19. However, a sign of the extraordinary commitment carried out by researchers around the world is provided by recently published scientific evidences. Suffice it to say that the word “SARS-CoV-2” is reported on PubMed in 45,860 scientific articles, with reference date as the end of December 2020, demonstrating how the scientific community has conducted intensive research on this emergent topic. However, it is also well known that the development of specific *de-novo* antiviral therapies generally takes from 10 to 17 years, while for particular vaccine production it should be necessary to wait for a timeframe of approximately 12–18 months.

In December 2020, the Food and Drug Administration (FDA) issued an Emergency Use Authorization (EUA) for the Pfizer-BioNTech COVID-19 (BNT162b2) vaccine (Pfizer, Inc; Philadelphia, Pennsylvania). This vaccine is a lipid nanoparticle-formulated nucleoside-modified mRNA vaccine encoding the prefusion Spike glycoprotein of SARS-CoV-2.

Despite the ultracold-chain storage and requirements for handling and administration, this vaccine will be feasible to implement. Still, these requirements could limit its availability to some populations, thereby negatively impacting health equity. In this perspective, scientific efforts should be made to overcome these challenges and advance health equity [4].

An issue to emphasize is the importance of the network between experts in complementary disciplines involved in COVID-19 investigation. In this connection, a trans-European Cooperation in Science and Technology (COST) action network is currently promoting scientific collaborations to speed up the achievements against COVID-19 [5].

Another main point is represented by the evolution and the adaptive mutations of the virus [6]. Indeed, the presence of SARS-CoV-2 Spike mutations can affect the interaction with the human epithelial cell receptors Angiotensin Converting Enzyme 2 (ACE2). The question of whether this would correspond to a novel and more potent COVID-19 specificities eventually requiring the development of wider-spectrum anti-SARS-CoV-2 strategies, such as vaccines or antiviral drugs, remains open [7,8,9].

Hence, the repurposing of clinically evaluated drugs is one of the most promising strategies to address the immense landscape of diseases, including infectious ones such as COVID-19. Remarkably, while most drug repurposing occurrences are based on synthetic compounds, naturally occurring molecules may offer significant opportunities. In fact, nature itself may be considered a magic bullet providing molecules with promising or still unexplored pharmacological properties [10,11]. It was reported that around 80% of the human population draws on traditional plants in the developing world for health quality and requirements [12,13]. Moreover, natural products are considered an essential source of successful drug leads and provide unique structural diversity with respect to standard combinatorial chemistry. Since more than 95% of the world’s biodiversity has not been evaluated for potential biological activity, the main challenge involves how to efficiently access and enhance this natural chemical diversity [14,15,16]. Therefore, the different chemical space structural, affordability, lack of substantial toxic effects and inherent biologic properties of natural products allow to consider them good candidates for new therapeutics [17,18]. As a result of the current COVID-19 outbreak, the repurposing of natural products could be an effective strategy to handle the SARS-CoV-2 infection. Currently, several studies have highlighted the antiviral properties of several natural compounds [12,19,20].

Therefore, naturally occurring molecules with broad antiviral spectrum may offer a safe, effective, and inexpensive platform for identifying novel treatment of SARS-CoV-2. To prepare this review article, the PubMed database (www.ncbi.nlm.nih.gov/pubmed) was used to analyze scientific manuscripts matching the queries “SARS-CoV-2” and “natural products”.

After analyzing the compelling articles dealing with the antiviral activity of natural compounds against SARS-CoV-2, we dedicated our attention to natural compounds recognizing SARS-CoV-2 druggable targets. Particularly, natural compounds currently under clinical trials were extensively herein discussed. Due to the amount of data from scientific literature, in our opinion sometimes inaccurate and superficial, our main goal is to give a vision on the possible role of the natural compounds against SARS-CoV-2.

In this regard, based on the recent analysis gathered on SARS-CoV-2 targets [21], we focused on natural compounds under clinical trials in the first section. Meanwhile, in the second section, we provided in-vitro, in-vivo and in-silico results of the natural derivatives against SARS-CoV-2. This analysis may help to figure out the most promising natural products which lead to the development of drugs for the treatment or prophylaxis of the emergent pandemic. Since the drug repurposing is a time-saving medicinal chemistry strategy, it could offer an excellent opportunity to obtain useful and non-toxic drugs to fight COVID-19.

## 2. Results

### 2.1. Natural Occurring Compounds Undergoing Clinical Trials

The clinical severity of COVID-19 varies from asymptomatic infection to mortal disease. Despite the academic and industrial efforts for fighting SARS-CoV-2, nowadays there are no drugs for the treatment of this epidemic viral infection. Hence, the development of effective and safe drugs is urgent to control or prevent the spread of SARS-CoV-2. Natural products, which have historically been used to avoid or alleviate several diseases, are still offering molecules with promising or yet unexplored pharmacological properties from the ancient time. Natural occurring compounds are among the options being considered for the treatment of SARS-CoV-2 infection as they are commonly inexpensive, available, and rarely have shown undesirable side effects [22]. In this section, we will discuss the natural compounds actually under clinical trials.

#### 2.1.1. Vitamin D

**Figure 1:**
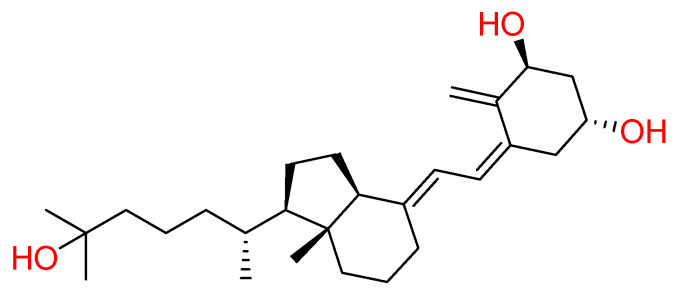
2D structure of calcitriol (Vitamin D).

In striving to determine a COVID-19 potential treatment, the effectiveness of **vitamin D** towards this endemic health issue was evaluated [23]. In ancient times, food-enriched vitamin D, including codfish, was used to prevent or treat acute respiratory syndrome [24]. Vitamin D is well-known to play an essential role in the maintenance of bone health and to regulate calcium-phosphorus metabolism [23]. Moreover, this vitamin is also responsible for the modulation of the immune response in infectious and autoimmune disease [25,26]. In particular, the active form of vitamin D, **calcitriol** has a defined role in immune function, with anti-inflammatory activity, inhibiting the overexpression of inflammatory cytokines (e.g., IL-1α, IL-1β, TNF-α) (Figure 1) [27].

Therefore, researchers have hypothesised a potential link between this compound and SARS-CoV-2, and vitamin D underwent several studies for evaluating its activity against COVID-19 [28].

A possible mechanism of action was attributed to the binding between the active form of vitamin D and S protein of SARS-CoV-2. Docking results showed that calcitriol recognised the Receptor Binding Domain (RBD) of Spike glycoprotein with good binding affinity, thus engaging hydrogen bonds with Gln493 residue and hydrophobic interactions with Ser494, Tyr449, Asn450, Leu452, Tyr489, Leu492, and Glu484 residues [29]. According to Weir et al., who recently reviewed vitamin D activities in the COVID-19 context, its supplement would offer an easy option in fighting against SARS-CoV-2 [30]. Indeed, the authors provided several reports demonstrating that low levels of vitamin D are associated with increased inflammatory cytokines, which contribute to worsening COVID-19 severity [30]. These reports raise the possibility that adequate vitamin D levels can reduce the severity of the cytokine storm, which can occur in COVID-19.

Interestingly, *Jain* and co-authors analysed the vitamin D level in both asymptomatic and critically ill COVID-19 patients and its correlation with inflammatory markers (IL-6; TNF-α, and serum ferritin) [31]. Vitamin D deficiency and serum level of inflammatory markers were higher in the patients with severe COVID-19 symptoms. Moreover, a positive correlation was found between the fatal rate and vitamin D deficiency [31]. Despite this evidence, which encourages a mass administration of vitamin D supplements to populations at risk of COVID-19, the study suffers some drawbacks. The current analysis has been conducted in a single centre located in India, an area with a high prevalence of vitamin D deficiency. It does not consider comorbidities, such as diabetes and hypertension [31].

Therefore, keeping these results in mind, a well-drawn multicentre study should be conducted to generate more robust conclusions. In general, retrospective studies demonstrated a direct link between vitamin D and COVID-19 cases and outcomes.

On the other hand, additional data did not prove this correlation [32,33,34]. Randomized-double-blind, large-scale, well-built studies are necessary to clarify the efficacy of vitamin D for SARS-CoV-2. As far as our knowledge goes on, no conclusive evidence underwent from clinical trials (www.clinicaltrials.gov).

#### 2.1.2. Vitamin C

**Figure 2:**
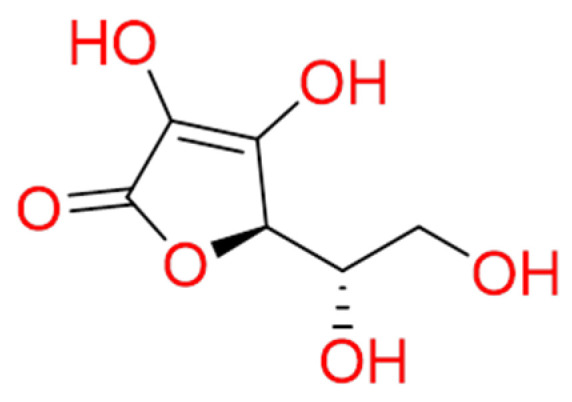
2D structure of ascorbic acid (Vitamin C).

**Vitamin C**, known as ascorbic acid, is an essential vitamin principally abundant in the *Citrus spp.* and commonly sold as a dietary supplement (Figure 2) [35]. Nowadays, it is under investigation for its biological effects for COVID-19 patients [36]. It has well-established antioxidant properties by scavenging oxygen free radicals and represents a key cofactor for the production of several neurotransmitters and hormones.

Vitamin C supports several cellular functions of both innate and adaptive immune systems [37,38]. Furthermore, upcoming studies demonstrated its anti-inflammatory properties, being able to inhibit pro-inflammatory mediators production, including IL-6, TNF-α, and the granulocyte-macrophage colony-stimulating factor (GM-CSF) signalling responses [39]. Altogether, the biological effects shown by vitamin C may help to inhibit the “cytokine storm” observed in the SARS-CoV-2 patients and to improve the host’s immunity. A meta-analysis of 29 clinical trials reported that vitamin C decreased the duration of the common cold by 8% in adults and 14% in children [40]. Furthermore, vitamin C ameliorated upper respiratory tract infections. However, its supplementation did not prevent the incidence of colds, indicating that a regular assumption is not justified [40].

Besides, the same authors reported in a meta-analysis of 12 clinical trials that vitamin C reduces the length of the intensive care unit stay by 7.8% and the duration of mechanical aided ventilation [41,42,43]. Emerging evidence, carried out on a small clinical trial with 54 critical COVID-19 patients, demonstrated that high dose vitamin C infusion decreased the IL-6 level, concluding that this therapeutical regimen may provide a protective clinical effect without any adverse events in critical COVID-19 patients [44]. Other outcomes were considered in the same clinical trial, including organ failure score, intensive care unit length, and mortality. According to the authors, a significant decrease in intensive care unit stay and hospital mortality was observed in the vitamin C treated patients [44]. Overall, vitamin C seemed to possess relevant pharmacological properties against severe respiratory infections, including COVID-19 [45,46]. Several clinical trials, assessing the efficacy of high dose vitamin C infusion, are still ongoing and will provide more definitive evidence.

#### 2.1.3. Lactoferrin

**Figure 3:**
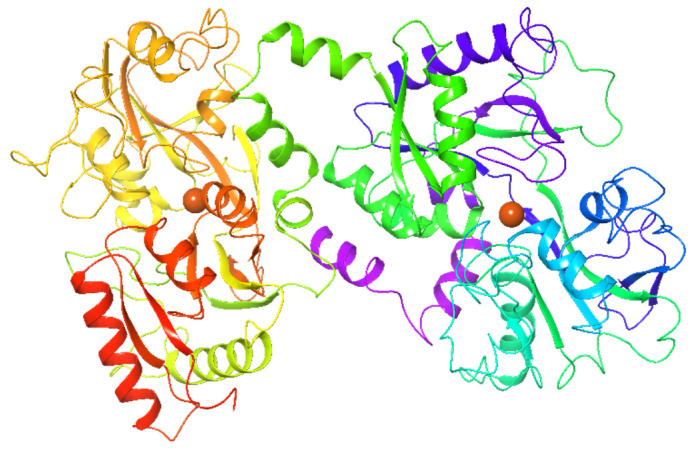
3D crystal structure human lactoferrin (PDB: 1B0L) [50]. Orange spheres represent Fe^3+^ cation binding sites.

**Lactoferrin (LF)**, or lactotransferrin, is a cationic glycoprotein constituted by 691 aminoacids folded into two globular lobes (80 kDa), connected by an α-helix. LF can reversibly chelate two Fe^3+^ cations per molecule (Figure 3) [47,48,49].

This protein is mainly produced by the mammalian gland, particularly in mammalian and animal milk [51]. When needed, LF may also be taken as a supplement, acting as a nutraceutical or functional food [52].

Due to its iron-binding capabilities, this protein regulates the iron homeostasis in the body and does not release iron even at pH 3.5 [53]. This property ensures iron sequestering in infected body compartments, where the pH is normally acidic, decreasing the availability of essential iron to microbes [53]. LF exert immune-modulatory effects by increasing the expression ability of B cells and by regulating the function of T cells [54]. Further, it is reported that LF has anti-inflammatory properties. This protein can access inside host cells and shift in the nucleus, thus regulating pro-inflammatory gene expression [54,55]. As an example, the down-regulation of IL-6 has been demonstrated in both in-vitro and in-vivo models [56,57].

LF is active against broad-spectrum viruses, including coronavirus [58,59]. It is believed that glycoprotein interacts with heparan sulfate proteoglycans of the host cells, preventing the entry of viral particles into host cells [60]. Therefore, LF has received the attention of the scientific community in the prevention of SARS-CoV-2 infection. Exploring the possible mechanisms of action involving LF, a computational investigation suggested its possible effect in the early stages of the infection. Indeed, by 2D-Zernike descriptors of molecular surfaces of sialic acid receptors on the host cell membrane, ACE2, S, M, and E proteins, it was highlighted that LF could affect the viral attachment to the host cell due to its probable competition with the virus in the binding to sialic acid [61,62,63,64]. Additionally, potential competition between ACE2 and LF in the binding site of SARS-CoV-2 Spike was suggested. This is probably due to two high complementarity regions in the *N-* and *C-* terminal domains of the Spike protein to LF [65].

Additional computational study sustained the idea of direct LF binding to the Spike S glycoprotein CDT1 domain, reducing the virus’s likelihood of entering cells. Moreover, after evaluating the human LF-Spike complex, it was observed that only two residues (Thr500 and Tyr505) are involved in the interface recognition [66].

From February 2020, the LF efficacy against SARS-CoV-2 has been reviewed since this protein showed promising antiviral and anti-inflammatory properties reducing the typical “cytokine storm” in COVID-19 patients [52,67,68]. Based on the encouraging preliminary results, several clinical trials have been registered but, the only clinical trial concluded was the one of Campione et al. [67]. According to the authors, LF might be used in asymptomatic or mildly symptomatic patients to prevent the worsening of SARS-CoV-2. However, despite the courageous preliminary results, more adequate and well-drawn clinical trials should be carried out to evaluate the useful, beneficial properties of LF for COVID-19 disease.

#### 2.1.4. Quercetin

**Figure 4:**
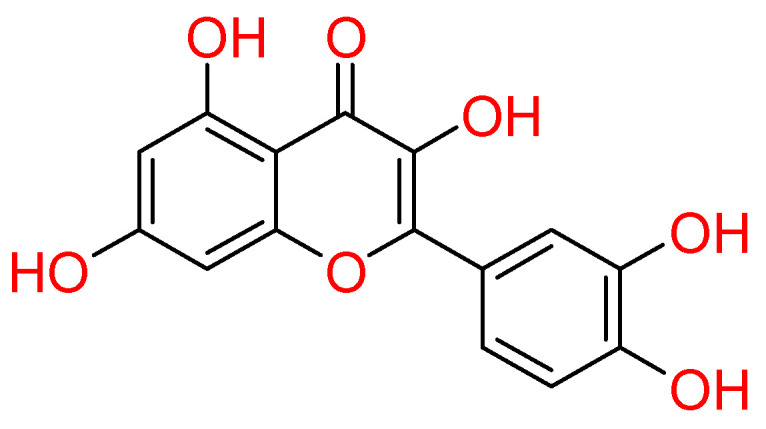
2D structure of quercetin.

**Quercetin** is a flavonol abundantly present in vegetables and fruits (Figure 4) [10].

This compound gained attention for its pharmacological properties, such as antiviral, anti-inflammatory, pro-metabolic and anticancer [10,69,70]. In light of this, quercetin was studied against COVID-19 as well.

In particular, the capability of quercetin to inhibit the proteases of SARS-CoV-2 was discussed. Regarding SARS-CoV-2 Mpro, docking studies highlighted quercetin as able to accommodate into this target binding site, establishing hydrogen bonds with Asn142, Ser144, and Met165. Also, hydrophobic contacts were established with Met49, Phe140, Leu141, and electrostatic interactions were engaged with His164 and Glu166 residues. The likelihood to interfere in the proteolytic activity of Mpro could be attributed to the destabilizing effect of quercetin into the catalytic pocket. It occurs due to its ability to adopt multiple binding poses, resulting in a favourable entropy gain. Strong experimental evidence validated this theoretical hypothesis [71]. Concerning the SARS-CoV-2 papain-like protease (PLpro), quercetin did not result as a good binder in a theoretical study [72]. Conversely, a computational investigation evidenced it as able to bind at S protein: ACE2 receptor interface [73]. In vitro studies demonstrated that quercetin inhibited recombinant human (rh) ACE2 activity [74,75]. More recently, the clinical trials (NCT04468139 and NCT04377789) including quercetin, were involved in both prophylaxis and treatment of COVID-19 [76,77,78].

#### 2.1.5. Resveratrol

**Figure 5:**
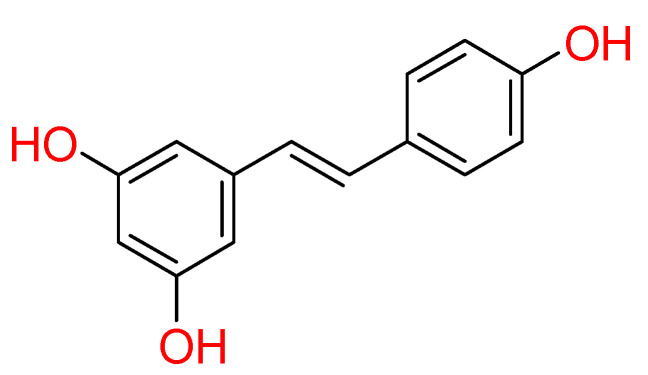
2D structure of resveratrol.

**Resveratrol** is the most extensively studied stilbene natural compound (Figure 5). Stilbenoids are phenolic molecules and due to their biosynthesis by the plants after ultraviolet radiation, toxins and injures, are also classified as phytoalexins. Chemically, stilbenoids show a common backbone including different types and position and substituents groups on the aromatic rings.

This kind of natural compound is endowed with numerous biological activities; therefore, recent studies focused on possible resveratrol effect against COVID-19 [17,79]. Computational analysis showed its good binding property towards SARS-CoV-2 Spike protein and human ACE2 receptor interface.

Docking results highlighted its strong interactions with crucial residues involved in the binding between S protein of SARS-CoV-2 and ACE2 receptor, establishing two hydrogen bonds with Lys353 of S protein and Gly496 of ACE2 receptor. The structural stability of resveratrol at its interface binding pose was confirmed by molecular dynamic simulations [80]. Furthermore, an in-vitro study reported a dose-dependent antiviral effect of resveratrol on SARS-CoV-2 infection in Vero E6 cells [81]. Currently, several clinical trials for resveratrol are undergoing (NCT04542993, NCT04536090, and NCT04377789).

#### 2.1.6. Hanfangchin A

**Figure 6:**
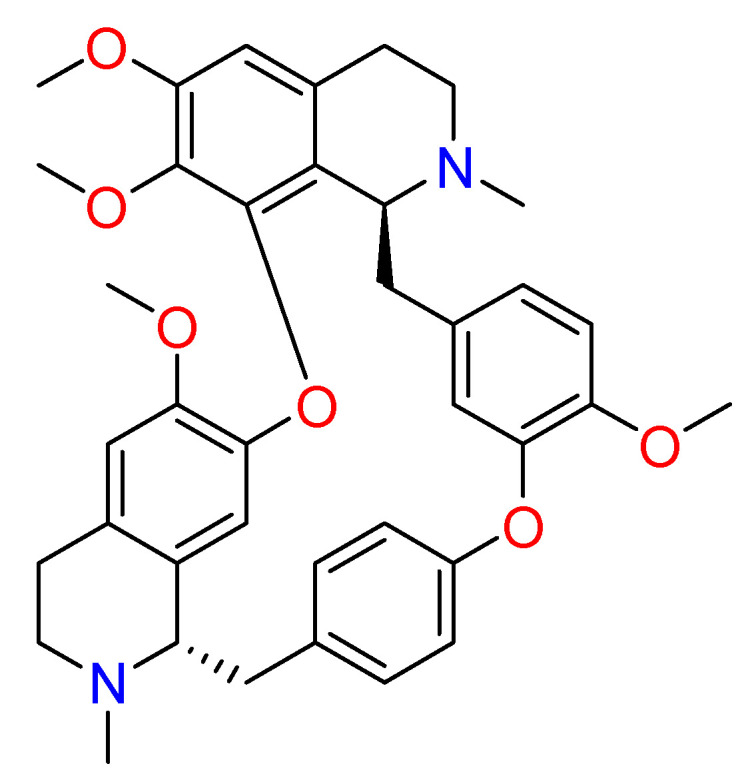
2D structure of hanfangchin A (tetrandrine).

According to Riva et al., **hanfangchin A**, also called tetrandrine (TET) (Figure 6), showed dose-dependent antiviral activities [82,83]. TET is a benzyltetrahydroisoquinoline alkaloid isolated from the root of the plant *Stephania tetrandra* with several pharmacological effects [84]. Structurally, two benzyl-tetrahydroisoquinolines are connected to ether linkages to form tetrandrine with tetrahydroisoquinoline *N*-methylation, bearing an irregular eighteen-member ring [85].

It was hypothesized that TET could work by blocking the two-pore channel 2 (TPC2) located in the membranes of host endolysosomal compartments by which SARS-CoV-2 can egress from these organelles and continue its replication [83,85].

TET was excluded by in silico studies because it violated three drug-likeness properties reported in Lipinski’s Rule of 5. In detail, it showed a LogP value of 6.4, and the increase of lipophilicity property can result in a probable lack of selectivity and attrition in drug development [86]. For this, further investigations are needed [87]. The promising pharmacological efficacy as anti-COVID-19 agent was demonstrated in Vero E6 (EC_50_ = 1.2 µM), Huh-7 (EC_50_ = 0.64 µM), and HEK293T (EC_50_ = 0.56 µM) cells transduced with ACE2. Furthermore, exceptional levels of synergy with remdesivir have been reported [88]. A clinical trial for TET has been designed (NCT04308317).

#### 2.1.7. Glycyrrhizin

**Figure 7:**
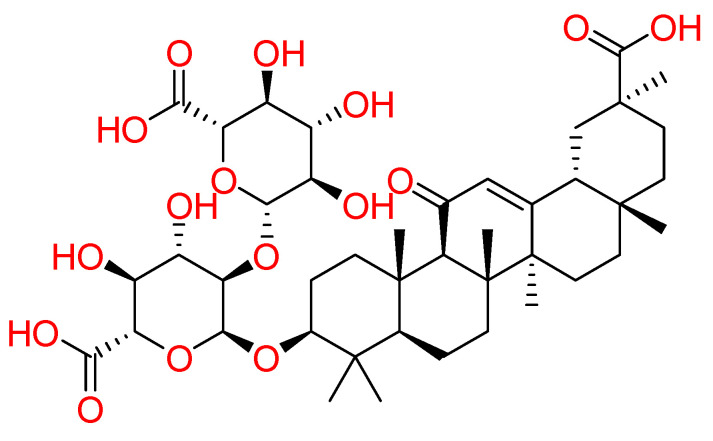
2D structure of glycyrrhizin.

**Glycyrrhizin**, an active component of liquorice roots, was investigated against SARS-CoV-2 (Figure 7). Combining high throughput screening (HTS) and computational approaches, for terpene-based compounds, it was analyzed the likelihood of Glycyrrhizin to insert between SARS-CoV-2 RBD-ACE2 complex. In detail, it is worth noting that Glycyrrhizin recognized Asn501, Gln498, Gly496, Tyr449, Tyr453, and Glu484 by means of hydrogen bond interactions and hydrophobic contacts with Tyr489, Phe456, and Leu455 [89]. Other glycyrrhizin involvements could be summarized as follows: (1) targeting S-RBD-ACE2 complex, (2) controlling pro-inflammatory cytokines, (3) preventing the accumulation of the intracellular ROS, (4) controlling thrombi, (5) preventing the fast production of airway exudates, and (6) persuading endogenous interferon against SARS-CoV-2 [90]. Recently, glycyrrhizin was recruited in a clinical trial (NCT04487964).

#### 2.1.8. Artemisinin

**Figure 8:**
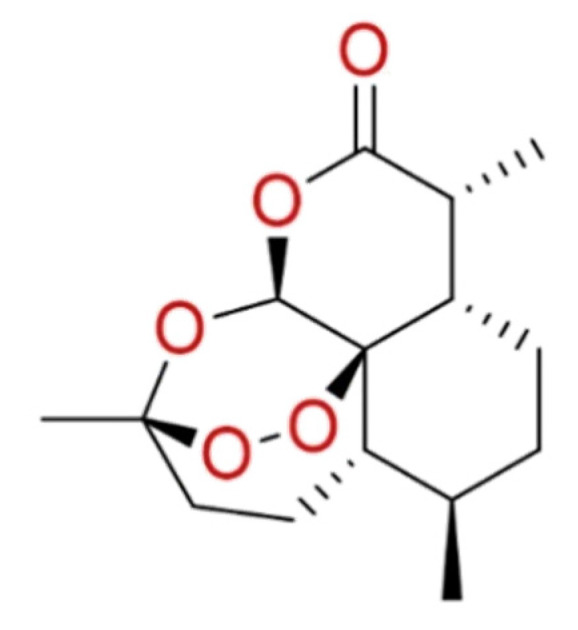
2D structure of artemisinin.

The anti-SARS-CoV-2 activity was also investigated for the compounds extracted from *Artemisia Annua*. Among these, **artemisinin** (Figure 8) the most abundant active component, is an antimalarial agent showing a promising broad-spectrum antiviral activity [91,92]. In-silico approaches helped to understand the possible mechanism of artemisinin to prevent the interaction between S protein and ACE2 receptor, due to its ability to bind Lys353 and Lys31 hotspot binding region [93].

To date, we have in-vitro evidence for artemisinin and its derivative arteannuin B, and a clinical trial is undergoing (NCT04382040) [93,94].

#### 2.1.9. Colchicine

**Figure 9:**
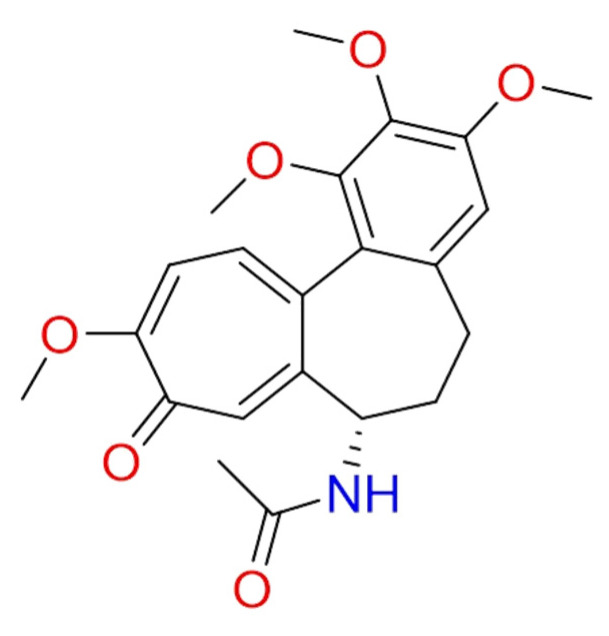
2D structure of colchicine.

**Colchicine** (Figure 9) extracted from the *Colchicum autumnale* is used for the treatment and management of patients with gout. In recent years, this old drug was also proposed in a variety of illnesses, including rheumatic and cardiovascular diseases [95].

The possible role of colchicine was hypothesized *versus* COVID-19 infection. Ito et al. highlighted how SARS-CoV and its accessory protein could activate pro-IL-1β gene transcription and protein maturation, and subsequently, NLRP3 inflammasome [96]. Indeed, the anti-inflammatory activity of colchicine mainly depends on the inhibition of the NLRP3 inflammasome [97]. Other anti-COVID-19 mechanism have been proposed [98].

Therefore, this compound could be utilized for the treatment of COVID-19. To date, several clinical trials are undergoing (NCT04527562, NCT04392141, NCT04375202, NCT04355143, and NCT04360980).

Scarsi et al., report a 20% improvement in the survival rate at 21 days in patients treated with colchicine compared with the local standard of care [99]. However, Gendelman and co-authors did not detect a potential benefit of colchicine for COVID-19 treatment [100].

#### 2.1.10. Berberine

**Figure 10:**
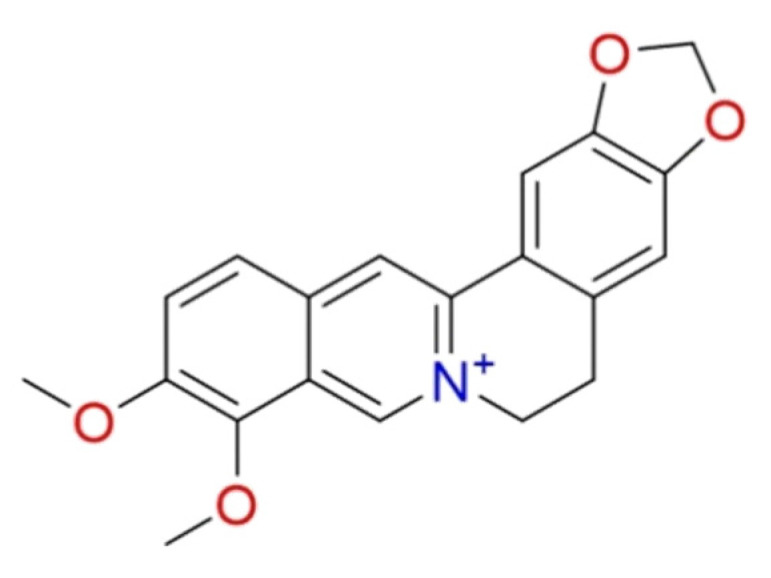
2D structure of berberine.

**Berberine** (Figure 10) is an alkaloid extracted from *Berberis vulgaris* L. which was extensively studied for its pharmacological activities with applications in several therapeutic areas, including cancer [101,102,103]. The antiviral activity of berberine against influenza and alphaviruses has been described [104]. In the SARS-CoV-2 context, Pizzorno et al. demonstrated the in vitro efficacy against SARS-CoV-2 in Vero E6 cells [105]. These findings agree with other studies [106,107]. An in silico study reported that berberine well-recognized the main protease by establishing hydrogen bond interactions with Phe140 and Asn142 [108].

Due to its promising anti-COVID-19 activities, this compound underwent in clinical trial, but no results are available (NCT04479202).

### 2.2. Natural Compounds with Promising Activities Against SARS-CoV-2 Infection

A few weeks later, after the outbreak of the pandemic, natural occurring compounds deriving from different chemical-diverse scaffolds showed promising data against SARS-CoV-2 druggable targets. However, as most scientific efforts provided computational studies without any experimental validation, the hope is that further in-vitro and in-vivo experiments will be performed. In this perspective, this section aims to summarize the results extrapolated by scientific literature achieved so far on the natural chemical scaffolds with promising activities against SARS-CoV-2 infection.

#### 2.2.1. Alkaloids

Alkaloids are fundamental chemical components representing a rich natural source for exploitation in human health. Their chemical scaffolds are characterized by nitrogen-containing groups, often within heterocyclic rings. Alkaloids show various biological properties in several diseases [109].

**Figure 11:**
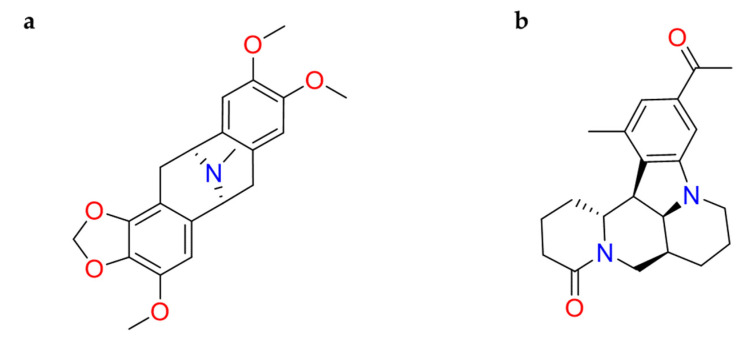
2D structures of (**a**) thalimonine and (**b**) sophaline.

Based on the known antiviral activity of some alkaloids, a computational study was designed to investigate their effect against SARS-CoV-2 Mpro. Among them, **thalimonine** and **sophaline** well recognized the binding pocket of the protease (Figure 11).

Thalimonine engaged two hydrogen bonds with Cys145 and Ser144 and a π-cation interaction with His41. Instead, sophaline was able to form a hydrogen bond with His163 and hydrophobic contacts with His41, Met49, Gly143, Cys145, Met165, and Gln189 residues.

**Figure 12:**
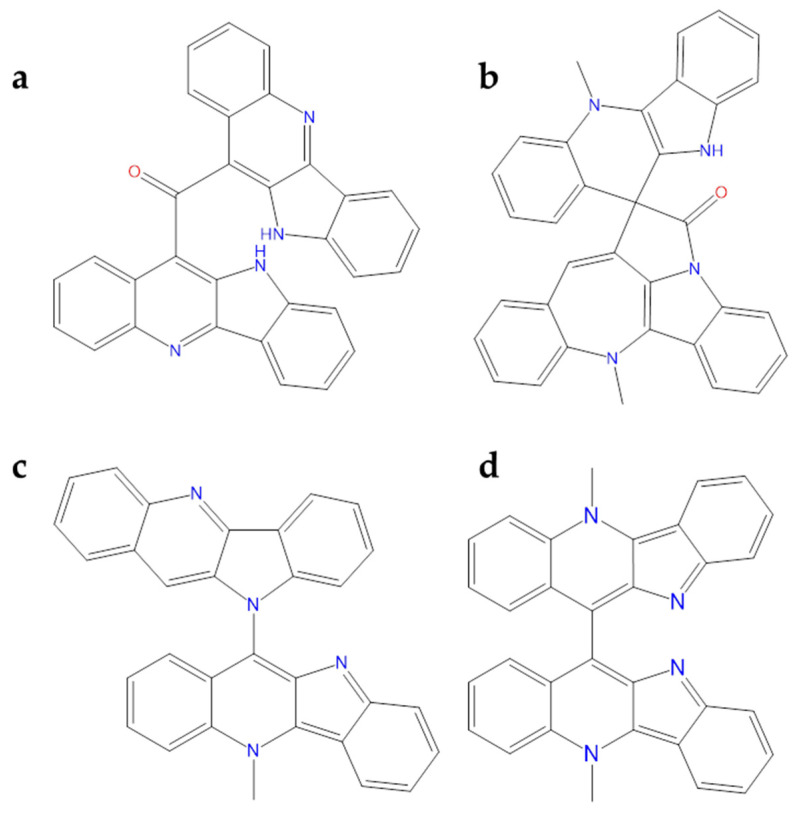
2D structures of (**a**) cryptomisrine, (**b**) cryptospirolepine, (**c**) cryptoquindoline, and (**d**) biscryptolepine.

Computational analysis of alkaloids from *Cryptolepis sanguinolenta* was performed against Mpro and RNA-dependent RNA–polymerase (RdRp), revealing that **cryptomisrine**, **cryptospirolepine**, **cryptoquindoline**, and **biscryptolepine** (Figure 12) exhibited robust binding affinity towards these two SARS-CoV-2 targets. Overall, all these alkaloids were able to recognize the active site of the two studied targets. Among these, for SARS-CoV-2 Mpro, **cryptospirolepine** well interacted with the catalytic dyad; for the polymerase, the best one was the **cryptomisrine** which was involved in *π*-cation interactions with Arg553 and Arg624 residues of SARS-CoV-2 RdRp [110]. Gyebi et al. studied the involvement of these alkaloids in the prevention of SARS-CoV-2 cell entry. In particular, **cryptospirolepine** was able to form several interactions with Phe40, Ala348, Arg393, His401 in the subdomain I of ACE2. In addition to the block access to ACE2, **cryptospirolepine** and **cryptoquindoline** could inhibit the cleavage of Spike glycoprotein by interacting with TMPRSS2 [111].

**Figure 13:**
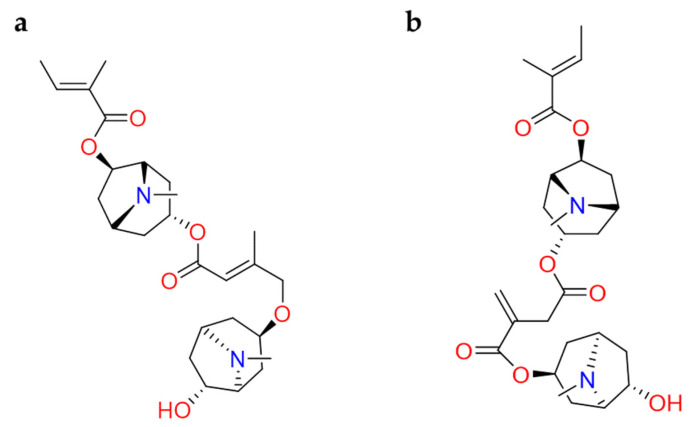
2D structures of (**a**) schizanthine Z and (**b**) schizanthine Y.

Also, tropane alkaloids from *Schizanthus porrigens* have been studied as promising inhibitors of SARS-CoV-2 PLpro. **Schizanthine Z** and **schizanthine Y** (Figure 13) were able to recognize the protein through the interaction with Tyr164 and Tyr173 residues; meanwhile, **schizanthine Y** engaged an additional hydrogen bond with Tyr168. The in-vitro experiments are highly recommended [112].

**Figure 14:**
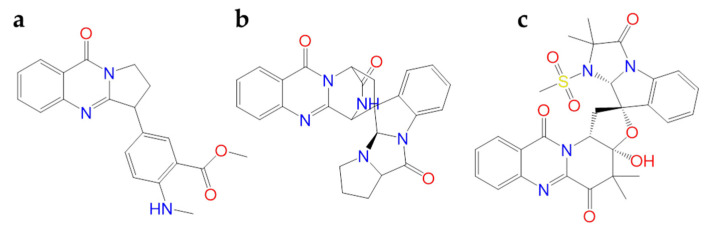
2D structures of (**a**) anisotine, (**b**) quinadoline B and (**c**) scedapin C.

Among the alkaloids from *Justicia adhatoda*, **anisotine** (Figure 14a) could have promising inhibitory activity against SARS-CoV-2 Mpro due to its interaction with the key catalytic residues (His41 and Cys145) of the Mpro pocket using computational approach [113].

Quimque et al. virtually screened the fungal natural products against PLpro and Mpro, RdRp, nsp15, and the Spike binding domain to glucose-regulated protein 78 (GRP78). It was resulted that two fumiquinazoline alkaloids **quinadoline B** and **scedapin C** (Figure 14b,c) had strong in silico inhibitory activity towards the analyzed SARS-CoV-2 targets [114].

#### 2.2.2. Terpenes

Recently, the involvement of plant-based terpenes was also noticed for COVID-19 infection [115,116]. Chemically, they are formed by multiple isoprene units (C5) and based on their number they are classified as hemi-, mono-, sesqui-, di-, tri-, tetra-, and polyterpenes. This class of chemical compounds along with their derivatives, terpenoids, which contains steroids/sterols, saponines and meroterpenes, offer an extensive range of applications in human health [117,118].

Recent investigations reported that **citronellol**, **geraniol**, **limonene**, **linalool**, and **neryl acetate** contained in the geranium and lemon essential oils down-regulated ACE2 receptor activity in virus-host epithelial cells [119]. Kulkarni et al. evaluated the major components of plant essential oils such as **anethole**, **cinnamaldehyde**, **carvacrol**, **geraniol**, **cinnamyl acetate**, **l-4-terpineol**, **thymol,** and **pulegone** as promising inhibitors of SARS-CoV-2 Spike protein by in-silico approach [120].

Among terpenoid derivatives, by molecular docking simulation, it was revealed that **limonin** and **scopadulcic acid B** showed a good binding affinity towards RdRp, Spike protein, and ACE2 receptor [119,121].

**Figure 15:**
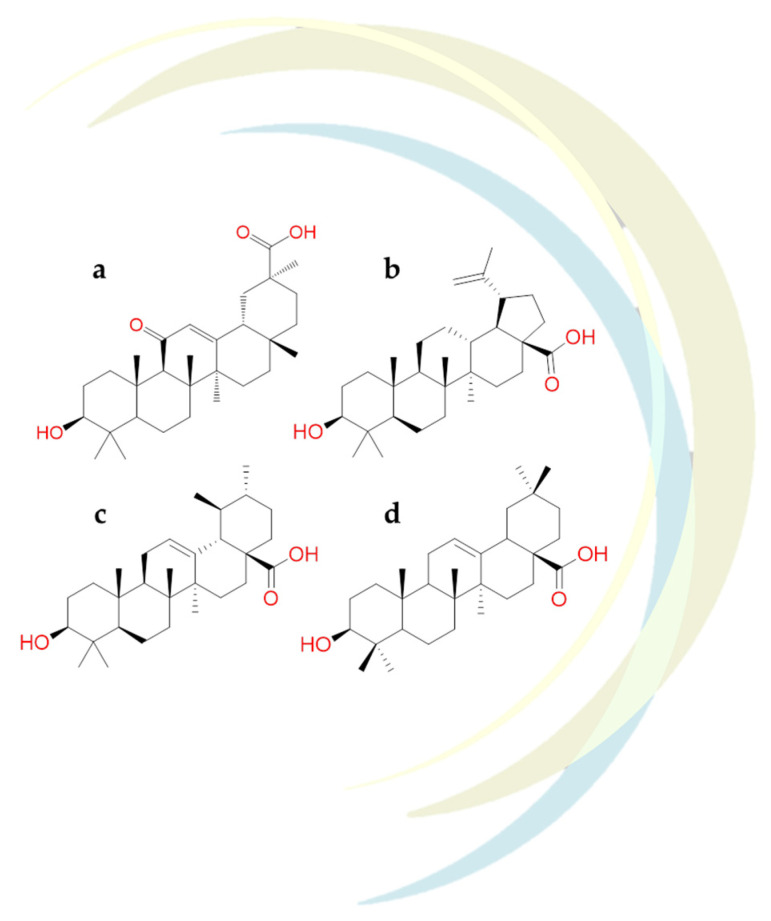
2D structures of (**a**) glycyrrhetinic acid, (**b**) betulinic acid, (**c**) ursolic acid, (**d**) oleanolic acid.

Carino et al. identified several triterpenoids and steroidal molecules (Figure 15) as inhibitors of Spike-ACE2 interactions [122]. In details, these studies reported that **glycyrrhetinic acid** (Figure 15a), **betulinic acid** (Figure 15b), and **oleanolic acid** (Figure 15d), were able to well recognize the RBD’s pocket 1. Docking results underlined that the steroidal scaffold could interact with Trp436, Phe374, Arg509, and Leu441 residues of the hydrophobic pocket of RBD. In addition, glycyrrhetinic acid engaged hydrogen bonds with Asn440, Ser375 and ionic contacts with Arg509.

The experimental results found that the incubation of the Spike-ACE2 complex with these naturally occurring triterpenoids reduced the Spike-ACE2 binding in a concentration-dependent way. Among all the compounds mentioned, **betulinic and oleanolic acid** showed significant inhibition at a concentration of 0.1 µM and 10 µM, respectively [122]. Interestingly, the pre-incubation of oleanolic and glycyrrhetinic acid with Spike-ACE2 complex exhibited a more remarkable ability to reduce the interaction between them and biological targets [122].

In-silico study revealed that **betulinic acid** and **glycyrrhetinic acid** (triterpene derivatives) showed good binding energies against the protease SARS-CoV-2. In detail, these compounds could recognize the Mpro structure pocket forming an hydrogen bonds with Lys137 and hydrophobic contacts with Tyr237, Tyr239, Leu272, Leu286, and Leu287 [123].

Based on the structural similarity between **oleanolic acid** and **ursolic acid** (Figure 15c,d)**,** and due to their comparable pharmacological activities, the possible binding mode of these compounds towards the catalytic pocket of SARS-CoV-2 Mpro was analyzed. Thus, it was observed that **ursolic acid** formed a hydrogen bond with Ser46 and several Van der Waals interactions with Thr24, Thr25, Thr26, Cys44, Thr45, Asn142, Gly143, Cys145, and Glu166 residues of the protease. Further, in silico results showed that **oleanolic acid** was involved with the catalytic dyad of the SARS-CoV-2 protease establishing both a hydrogen bond and several hydrophobic contacts with Gln189, Cys145 and His163 residues, respectively [124].

**Figure 16:**
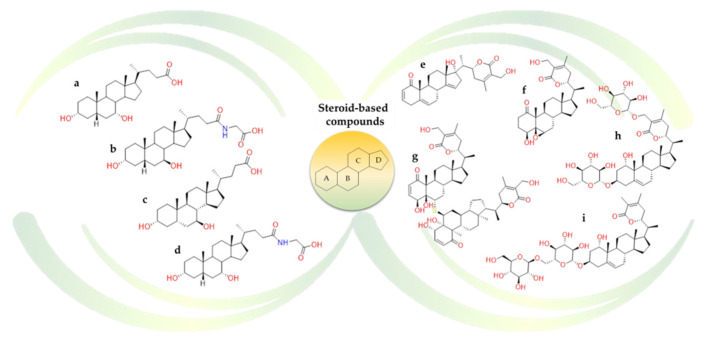
2D structures of (**a**) chenodeoxycholic acid, (**b**) ursodeoxycholic acid, (**c**) Glyco-ursodeoxycholic acid, (**d**) glyco-chenodeoxycholic acid, (**e**) withanolide N, (**f**) dihydrowithaferin A, (**g**) ashwagandhanolide, (**h**) withanoside X, (i) withanoside V.

According to the idea that these triterpenoids are the natural substrates for two bile acid receptors such as Farnesoid-X-Receptor (FXR) and G-protein Bile Acid Receptor (GPBAR)-1, the behaviour of some natural **bile acids** (Figure 16) were also investigated against SARS-CoV-2 druggable targets. The analyzed steroidal compounds were able to fit into pocket 5 on the surface of the β-sheet core of Spike RBD. In detail, the steroidal scaffold of the **ursodeoxycholic acid** (Figure 16b) is surrounded by Lys378, Thr376, Phe377, Tyr380, and Pro384 and formed a hydrogen bond with Cys379 [122]. The in vitro screening confirmed that the **ursodeoxycholic acid** and its taurine conjugate exerted a limited inhibitory activity towards Spike-ACE2 complex. In contrast, **glyco-ursodeoxycholic acid** (Figure 16c)**, chenodeoxycholic acid** (Figure 16a)**,** and **glyco-chenodeoxycholic acid** (Figure 16d) reduce the Spike binding to the ACE2 receptor by at least 20% in a concentration-dependent manner. Despite these promising in-vitro results, the studies have several limitations; e.g., the effects of these natural molecules were not tested on viral replication [122].

In addition to the activity against ACE2, the binding affinity of triterpene derivatives and steroid-based compounds against SARS-CoV-2 Mpro was explored. The **ursodeoxycholic acid** formed two hydrogen bonds with Phe140 and Ser46 residues, while Met49, Met165, Cys145, and Leu141 residues participated in hydrophobic interactions that enhanced stability of the protein-ligand complex. These in silico results showed that ursodeoxycholic acid had potential viral inhibitory activity towards SARS-CoV-2 Mpro and human ACE2 protein, but further experimental validations are needed to confirm their biological activity [125].

Additionally, several natural constituents of *Withania somnifera*, also known as Ashwagandha and “Indian Ginseng”, were explored as possible inhibitors of Mpro SARS-CoV-2 protease. From the study, **withanoside V** (Figure 16i), a steroid-based compound, was the most potent natural inhibitor [126].

**Figure 17:**
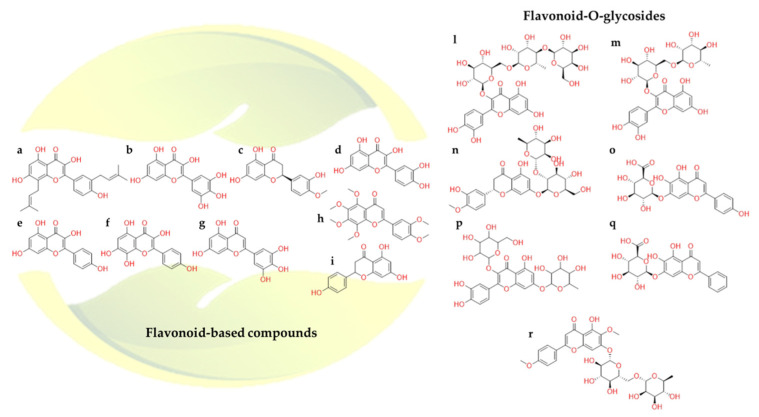
2D structures of (**a**) broussoflavonol F, (**b**) myricetin, (**c**) hesperetin, (**d**) quercetin, (**e**) kaempferol, (**f**) herbacetin, (**g**) tricetin, (**h**) nobiletin, (**i**) naringenin, (**l**) quercetin-3-*O*-galactosyl-rhamnosyl-glucoside, (**m**) rutin, (**n**) neohesperidin, (**o**) scutellarin, (**p**) quercetin-3-β-galactoside, (**q**) baicalin, (**r**) pectolinarin.

Others phytoconstituents from *Withania somnifera* such as **quercetin-3-*O*-galactosyl-rhamnosyl-glucoside** (Figure 17l), **withanoside X** (Figure 16h), **ashwagandhanolide** (Figure 16g), **dihydrowithaferin A** (Figure 16f) and **withanolide N** (Figure 16e) were investigated as promising inhibitors of Spike glycoprotein and Nsp15 endoribonuclease [127].

Among phytoconstituents from *Nigella sativa*, **campesterol**, **cycloeucalenol**, **apha-spinasterol,** and **beta-sitosterol** showed strong affinities against viral N-terminal RNA binding domain (NRBD) and PLpro of SARS-CoV-2 [128].

#### 2.2.3. Flavonoids

Among the naturally occurring compounds and beyond, flavonoids (Figure 17) are particularly studied [129], including their role versus SARS-CoV-2 [130]. Many in-silico screenings of medicinal plant databases against SARS-CoV-2 druggable targets have been carried out [131,132]. Indeed, analyzing the potential role of the flavonoids against SARS-CoV-2 main protease [133], it was noticed that their inhibitory activity was more extensive than that of the peptide-derived inhibitors [134]. Starting from this *rationale*, a thorough investigation was focused on the common pharmacophoric features of flavonoids.

The presence of two phenyl groups could be responsible for inhibiting the proteolytic activity of SARS-CoV-2 protease. In particular, by analyzing docking and molecular dynamics results, the hydrogen bond between the hydroxyl group of phenyl moiety of **kaempferol** (Figure 17e) and Glu166 was formed; meanwhile, the chromen-4-one scaffold was able to accommodate into the hydrophobic site. Additionally, the complex was further stabilized by two hydrogen bonds between the hydroxyl groups and Ile188 and Asp142 [134,135].

Again, **quercetin-3-β-galactoside** (Figure 17p) a flavonoid-based compound, was found to bind SARS-CoV-2 Mpro through numerous interactions with the key residues of the catalytic pocket. Notably, the *N* atom of the main chain of Glu166 made two hydrogen bonds with the compound, while the Gln189 side chain was able to form other four similar interactions. The hydrophobic contacts between the compound and Leu141, Asn142, Met165, and Glu166 residues were evident too. Concentrating on their chemical scaffold, it is possible to rationalize that: (1) four hydroxyl groups of quercetin are crucial determinants of the bioactivity of this type of compounds; (2) the shape of sugar moiety and 7-hydroxy site of quercetin can be characterized by large structural modifications in order to enhance the binding interactions [136].

Other examples were represented by the computational analysis performed on **baicalin** (Figure 17q), **herbacetin** (Figure 17f), and **pectolinarin** (Figure 17r) towards SARS-CoV-2 Mpro. Therefore, herbacetin showed the same kaempferol binding mode. Pectolinarin showed its l-mannopyranosyl-β-d-glucopyranoside moiety accommodated into the S1 and S2 sites and the chromen-4-one scaffold located within the S2 and S3’ SARS-CoV Mpro domains. The baicalin glucuronate moiety interacted through two hydrogen bonds with Gly143 and Asn142 residues. In addition, the *π-π* stacking between His41 and its phenyl moiety was observed. In this case, Glu166 plays a pivotal role in the binding mode due to its interaction with the 6-hydroxyl group attached to the chromen-4-one moiety and the 5-hydroxyl group attached to the glucuronate moiety. Baicalin, herbacetin, and pectolinarin revealed the prominent inhibitory activity against SARS-CoV-2 Mpro. Indeed, the experimental IC_50_ values were 34.71, 53.90, and 51.64 µM, respectively [135].

In-silico study reported that **broussoflavonol F** (Figure 17a) could better recognize SARS-CoV-2 Mpro active site due to the interaction with the catalytic dyad Cys145 and His41 [137].

In addition to the estimated binding affinity towards the protease, flavonoids have also shown promising ACE2 inhibitory activities [138]. A study highlighted that **quercetin** (Figure 17d), **myricetin** (Figure 17b), **hesperetin** (Figure 17c), **tricetin** (Figure 17g), and **rutin** (Figure 17m) presented stable binding affinity against Spike-ACE2 protein [132]. It was suggested that these compounds formed interactions with Arg273, Thr371, His345, Pro346, Glu375, Glu402, and Tyr515 in the ACE2 binding site. With this in mind, it was hypothesized that these natural compounds could compete with the bind of SARS-CoV-2 to ACE2, thus preventing the virus entry into host cells [139,140].

Furthermore, other research team noticed that, in addition to the above-mentioned flavonoids, **nobiletin** (Figure 17h), **naringenin** (Figure 17i), **neohesperidin** (Figure 17n), and **scutellarin** (Figure 17o), were also able to interact with the Spike-ACE2 complex [138]. However, their IC_50_ values were not still calculated. Despite the encouraging results of these natural compounds, in vitro steps were halted. Some drawbacks probably related to the extraction of these compounds from plants or the chemical synthesis difficulties did not permit further biological investigations. Recently, in-vitro study revealed that neohesperidin could inhibit infected Vero E6 cell [141].

Baicalin interacted at the interface between ACE2 receptor and Spike protein through Asp67, Ala71, Lys74, and Asn448, Ala464, Val472 and Gly474 residues, respectively. These in silico results were validated by in vitro experiments, having an IC_50_ around 2.24 µM on inhibiting the Spike protein and ACE2 receptor binding [138].

#### 2.2.4. Benzoquinones

Quinones are a fascinating class of compounds with attractive chemistry. 1,4-benzoquinones reoccurring structural motif is present in various isolated natural products, and this bestowing a broad range of biological activities [142]. Additionally, their derivatives also have shown promising ACE2 inhibitory activities [138].

**Figure 18:**
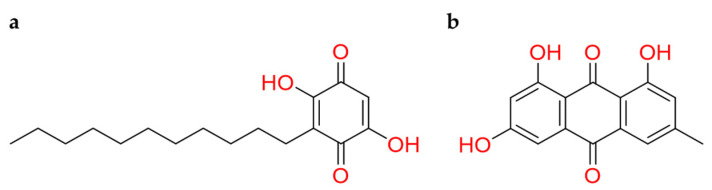
2D structures of (**a**) embelin and (**b**) emodin.

In this regard, **embelin**, a naturally occurring para-benzoquinone extract from *Embelia ribes* plant, aroused particular interest (Figure 18a). This natural compound showed several pharmacological effects, and it was considered the second solid gold of India, preceded by curcumin [143,144]. The *rationale* is addressable to the quinone reactivity with cysteine protease [145]. Moreover, a density functional theory (DFT) computational approach highlighted the mechanism of action of embelin into SARS-CoV-2 protease. The covalent bond between the quinone carbonyl and the thiolate of Cys145 was hypothesized. To help this reaction mechanism, His41 captured the hydrogen atom from Cys145 that became prone to release a proton to the carbonyl quinone engaged with the thiolate [146].

By computational methods was observed that **emodin** (Figure 18b) binds Asp67, Ala71, and Lys74 residues of ACE2 receptor, and Asn448, Ala464, Val472, and Gly474 residues of Spike protein [147]. An experimental study has been shown that emodin, extracted from *Rheum officinale* and *Reynoutria multiflora*, could act as an inhibitor of the viral Spike protein and human ACE2 receptor binding with IC_50_ value approximately of 1–10µM in other SARS coronavirus [148]. Thus, its promising theoretical mechanism and its known experimental activity against other coronaviruses can stimulate to investigate the potential inhibitory effect against SARS-CoV-2.

#### 2.2.5. Other Natural Compounds

In-silico studies were also performed on natural compounds that are readily available in onion, garlic, ginger, peppermint, and fenugreek [149,150]. The computational results pointed out **leucopelargonidin** and **aronadendin** are promising Mpro inhibitors, while **5S-5-Hydroxy-1,7-bis(4-hydroxy-3-methoxyphenyl)-3-heptanone**, contained in Ginger, interacted with the SARS-CoV-2 Spike protein [111,132]. This study needs appropriate biological experimental validation against COVID-19 [119,121].

Among phenolic compounds extracted from honey and propolis, it was observed that **ellagic acid** made hydrogen bonds with Gly808, Pro809, His816, thr817 and Tyr831 residues of SARS-CoV-2 RdRp. Docking results also showed that hydrogen bonds between ellagic acid and His41, Gly143, Arg188 were formed. Furthermore, Shaldam et al. proposed **artepillin C** as a potential inhibitor of the SARS-CoV-2 Mpro due to the interactions between the phenolic compound and Cys145, Arg188, Thr190 and Gln192 [151].

## 3. Discussion and Future Perspectives

Since SARS-CoV-2 has become a pandemic infection with relevant health issues, the scientific community carried out much effort for the identification of potential drug targets to tackle this virus infection. Recently, several articles have been published, most of them based on the virtual screening of natural occurring compounds towards viral target proteins [152,153,154,155].

Especially in emergent diseases, such as COVID-19, computer-assisted techniques are useful tools to find out potential new drugs. By harnessing the innovative technologies in rational drug design in the medicinal chemistry field, it has been possible to obtain promising compounds fitted to recognize several targets of the other coronaviruses [21,156].

Several chemical entities have already been investigated by computational approaches such as docking, DFT, molecular dynamics simulations; however, no novel compounds have been proposed until now, except for repurposed drugs.

Considering this, drug repurposing represents the promising strategy to fight tricky illness emerging in the future. Combined with polypharmacology, as a new therapeutic paradigm, the efforts of the scientific research towards the natural source compounds, though not new, could offer an opportunity to obtain the natural drugs scaffold useful against SARS-CoV-2 targets [157]. Naturally occurring compounds recognizing SARS-CoV-2 druggable targets are reported in Table 1.

Summarizing, numerous vitamin D clinical trials for COVID-19 could be useful to determine its effect on disease progression and post-exposure prophylaxis. One idea is that its supplementation can help patients to maintain sufficient serum levels of vitamin D as recommended by guidelines [160]. The known potential of vitamin D modulating the innate immune response may be auspicious to prevent acute respiratory infection [161]. Based on the knowledge that COVID-19 disproportionately affects race, ethnicity, minority groups, and the known vitamin D deficiency in the same groups, the use of natural derivatives may provide a chance for decreasing health disparities [162].

LF is the naturally occurring compound mostly differing from the others. Clinical trials are designed to evaluate the local treatment of nasal mucosa with LF by using a spray formulation. The *rationale* of the implication of LF for COVID-19 prevention has the following fundaments: reverting the iron disorders due to the viral colonization, modulating the immune response or down-regulating the pro-inflammatory cytokines released by the viral inflammation [67].

Among the flavonoid-based compounds, only quercetin is undergoing clinical trial due to its anti-inflammatory and scavenging activity. In detail, its dose scheduled is 500–1000 mg for prophylaxis and treatment, respectively [78].

Quercetin, in combination with zinc, bromelain and vitamin C, is recruited in clinical trials [77]. Several clinical trials on resveratrol, artemisinin, glycyrrhizin, colchicine, berberine, and tetrandrine have been designed and are going on.

These compounds are characterized by well-known beneficial activities other than the antiviral implications against COVID-19 above-mentioned. Since these components are already taken differently in the daily diet, it could be challenging to attribute the antiviral properties to these natural compounds directly. Still, their assumption could be valuable for human health protection and to positively fill health equity.

Herein, terpenes, anthraquinones, flavonoids, stilbenes, steroid-based scaffold, quinone derivatives, and polyphenols have been summarized based on their current update in the recognition and validation of the SARS-CoV-2 druggable targets.

Although some of these have several drawbacks and lead to inconsistent results [2,163,164,165,166,167,168,169,170]. In this regard, we would like to provide critical thought. Undoubtedly, the computational tools are valid instruments for the drug identification process, but the support of these preliminary investigations with experimental biological data are strictly required. Although these feelings can appear obvious, we believe this concept should be stressed to avoid the spread of disinformation or to prevent equivocal scientific conclusions. Most investigations were carried out on therapeutic benefits of phytochemicals, according to their known antiviral, anti-inflammatory and immunoregulatory properties. Several research teams conclude by asserting that the use of polyphenolic compounds, anthraquinones and flavonoids, and steroid-based molecules for COVID-19 treatment can appear speculative. In fact, no clear evidence from well-drawn clinical trials has been reported so far. On the other hand, dietary use of these phytochemical derivatives is known to provide several benefits to humans. The safety and efficacy of the reported natural compounds in preclinical and clinical trials must be still assessed to establish their use and application in COVID-19 infection. In this perspective, the dietary nature and pleiotropic effects make natural occurring compounds a fascinating candidate for further investigation.

Moreover, rational drug design highlights the possible binding mode of these compounds towards the key anti-SARS-CoV-2 targets. These data could be useful to suggest structural chemical modifications to improve the in-silico binding affinity. We expect scientists to perform relevant experimental studies to provide new derivatives within COVID-19 prevention and treatment, starting from the appropriate and most promising natural scaffolds.

## Figures and Tables

**Table 1:** Naturally occurring compounds recognizing SARS-CoV-2 druggable targets under investigation.

Compound Name	Natural Sources	In Silico	In Vitro	Clinical Trials	Ref
Alpha-spinasterol	*Nigella sativa*	•			[128]
Anethole	*Fennel*, *anise*, *Star anise*, *Essential oils*	•			[120]
Anisotine	*Justicia adhatoda*	•			[113]
Aronadendin	*Onion*	•			[132]
Arteannuin B	*Artemisia annua*		•		[94]
Artemisinin	*Artemisia Annua*	•	•	•	[93,94,158]
Artepillin C	*Honey and propolis*	•			[151]
Ashwagandhanolide	*Withania somnifera*	•			[127]
Baicalin	*Scutellaria baicalensis*	•	•		[135,138]
Berberine	*Berberis vulgaris* L.	•	•	•	[101,102,104,105,106,107,108]
Beta-sitosterol	*Nigella sativa*	•			[128]
Betulinic Acid	*Betula pubescens*	•			[123]
Biscryptolepine	*Cryptolepis sanguinolenta*	•			[110]
Broussoflavonol F	*Macaranga indica*	•			[137]
Campesterol	*Nigella sativa*	•			[128]
Carvacrol	*Origanum vulgare*	•	•		[120]
Chenodeoxycholic acid	*Bile Acids*	•	•		[122]
Cinnamaldehyde	*Cinnamomum* sp.	•			[120]
Cinnamyl Acetate	*Essential oils*	•			[120]
Citronellol	*Cymbopogon winterianus*, *Geranium*	•	•		[119]
Colchicine	*Colchicum autumnale*			•	[99,100]
Cryptomisrine	*Cryptolepis sanguinolenta*	•			[110]
Cryptoquindoline	*Cryptolepis sanguinolenta*	•			[110]
Cryptospirolepine	*Cryptolepis sanguinolenta*	•			[110]
Cycloeucalenol	*Nigella sativa*	•			[128]
Dihydrowithaferin A	*Withania somnifera*	•			[127]
Ellagic acid	*Honey and propolis*	•			[151]
Embelin	*Embelia ribes plant*	•			[146]
Emodin	*Rheum officinale* and *Reynoutria multiflora*	•	•		[147,148]
Geraniol	*Essential oils*	•	•		[119,120]
Glico-chenodeoxycholic acid	*Bile Acids*	•			[122]
Glyco-ursodeoxycholic acid	*Bile Acids*	•	•		[122]
Glycyrrhetinic Acid	*Glycyrrhiza glabra*	•			[123]
Glycyrrhizin	*Glycyrrhiza glabra*	•	•	•	[89,90]
Hanfangchin A	*Stephania tetrandra*	•	•	•	[82,83,84,88,159]
Herbacetin	*Rhodiola rosea*	•	•		[135]
Hesperetin	*Citrus* sp.	•			[140,141]
Kaempferol	*Aloe vera*	•			[134]
L-4-terpineol	*Essential oils*	•			[120]
Lactoferrin	*Mammalian and Animal milk*	•		•	[56,57,67]
Leucopelargonidin	*Onion*	•			[111,132]
Limonene	*Essential oils*	•	•		[119]
Limonin	*Euodia rutaecarpa*	•			[119,120,121]
Linalool	*Essential oils*	•	•		[119]
Myricetin	*Onion*, *Chilli*	•			[132,139,140]
Naringenin	*Citrus* sp.	•			[138,139]
Neohesperidin	*Citrus* sp.	•	•		[138,139,141]
Neryl Acetate	*Citrus oils*	•	•		[119]
Nobiletin	*Citrus* sp.	•			[138,139]
Oleanolic Acid	*Olea europaea* L.	•	•		[122]
Pectolinarin	*Cirsium setidens*	•	•		[135]
Pulegone	*Essential oils*	•			[120]
Quercetin	*Onion*, *Garlic*, *Peppermint*, *Fenugreek*, *Origan* *Capers*, *Onions*, *Elderberries*, *Kale*, *Okra*, *Apple Peels*, *Aronia Berries*, *Cranberries*, *Asparagus*, *Goji Berries*	•	•	•	[10,71,132,139]
Quercetin-3-O-galactosyl-rhamnosyl-glucoside	*Withania somnifera*	•			[127]
Quercetin-3-β-galactoside	*Artemisia capillaris*	•			[136]
Quinadoline B	*Fungi*	•			[114]
Resveratrol	*Red wine*, *Red grape juice*, *Peanuts*, *Fresh grapes*, *Pistachios*, *Peanut butter*, *Cocoa powder*, *Dark chocolate*, *Milk chocolate*, *Strawberries*, *Jackfruit skin*, *Blueberries*, *Bilberries*, *Red currants*, *Cranberries*, *Lingonberries*, *Mulberries*	•	•	•	[80,81]
Rutin	*Citrus* sp.	•			[139]
Scedapin C	*Fungi*	•			[114]
Schizanthine Y	*Schizanthus porrigens*	•			[112]
Schizanthine Z	*Schizanthus porrigens*	•			[112]
Scopadulcic Acid B	*Scoparia dulcis*	•			
Scutellarin	*Scutellaria barbata*	•			[139]
Sophaline	*Sophora alopecuriodes*	•			[86]
Thalimonine	*Thalictrum simplex*	•			[86]
Thymol	*Essential oils*	•			[120]
Tricetin	*Ginger*, *Onion*, *flowers of pomegranate.*	•			[132]
Ursodeoxycholic acid	*Bile Acids*	•	•		[122]
Ursolic acid	*Bile Acids*	•	•		[122]
Vitamin C	*Citrus Fruit*, *Tropical Fruit*, *Peppers*, *Cruciferous Vegetables*, *Leafy Vegetables*			•	[36,40,41,42,43,44,45,46]
Vitamin D	*Human and Jay Jasmine plant*	•		•	[32,33,34]
Withanolide N	*Withania somnifera*	•			[127]
Withanoside V	*Withania somnifera*	•			[127]
Withanoside X	*Withania somnifera*	•			[127]
5*S*-5-Hydroxy-1,7-bis(4-hydroxy-3-methoxyphenyl)-3-heptanone	*Ginger*	•			[132]
